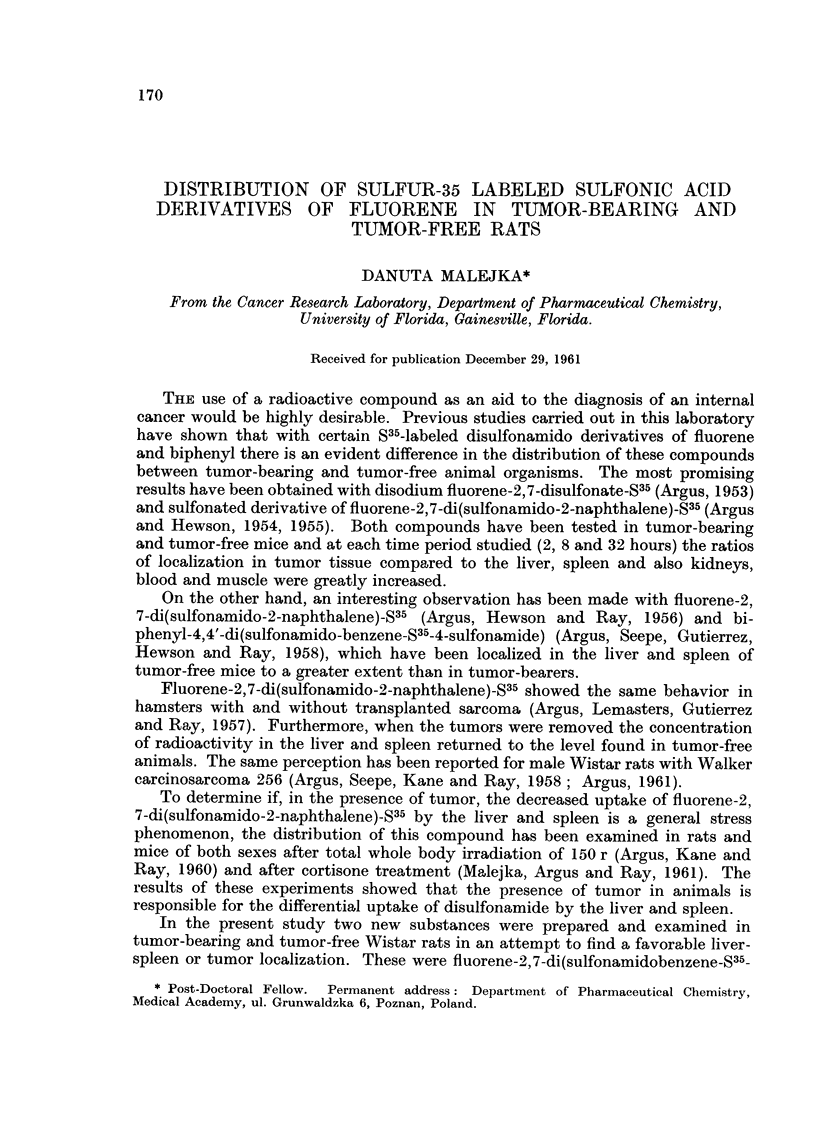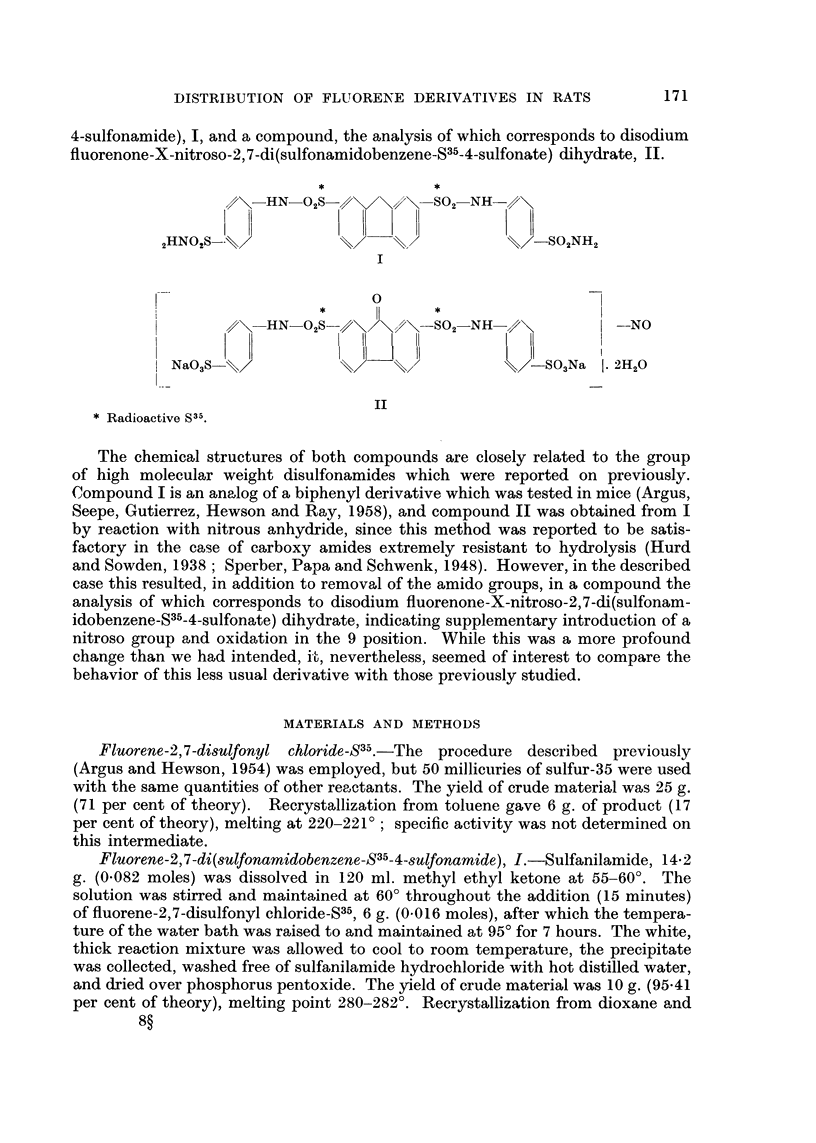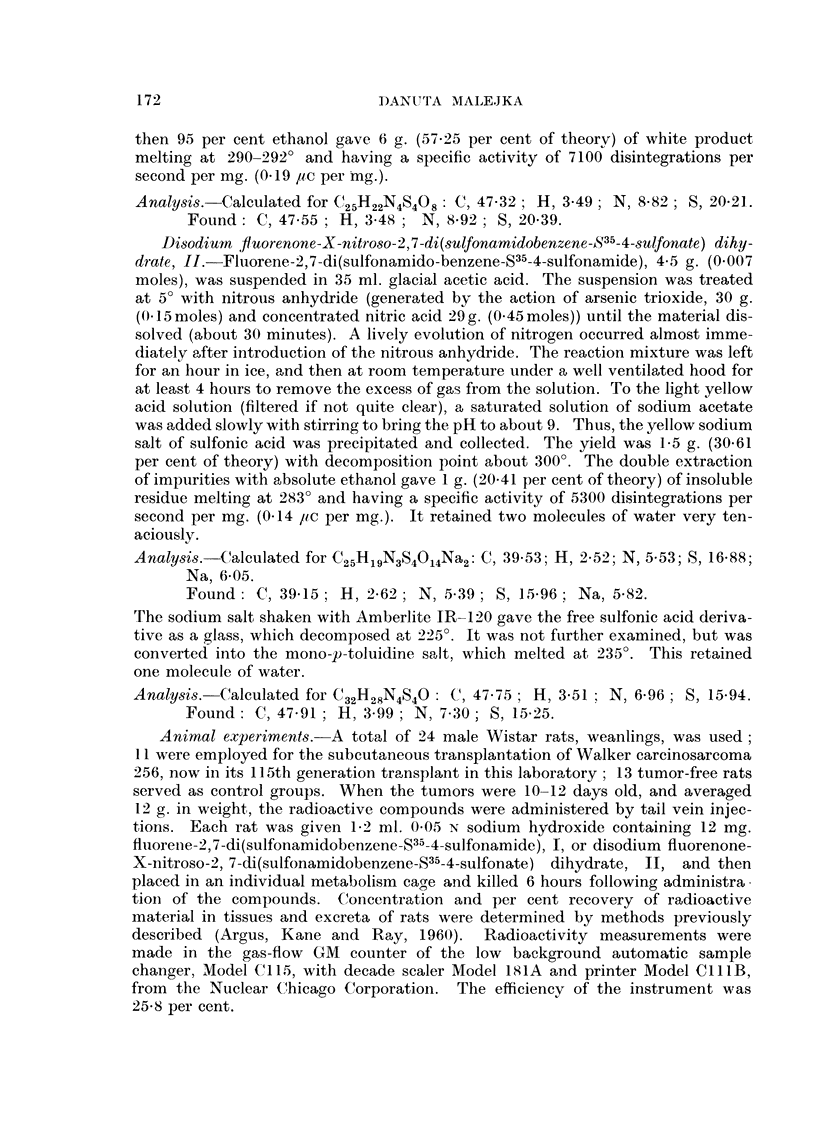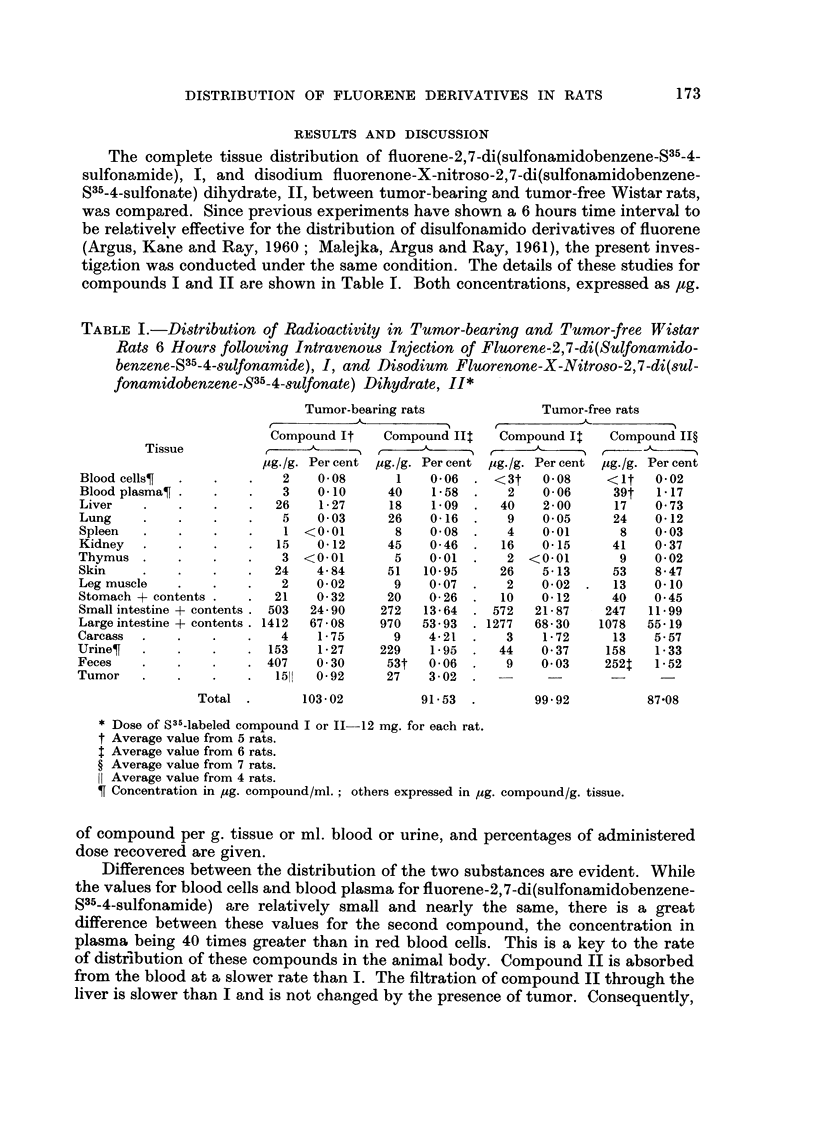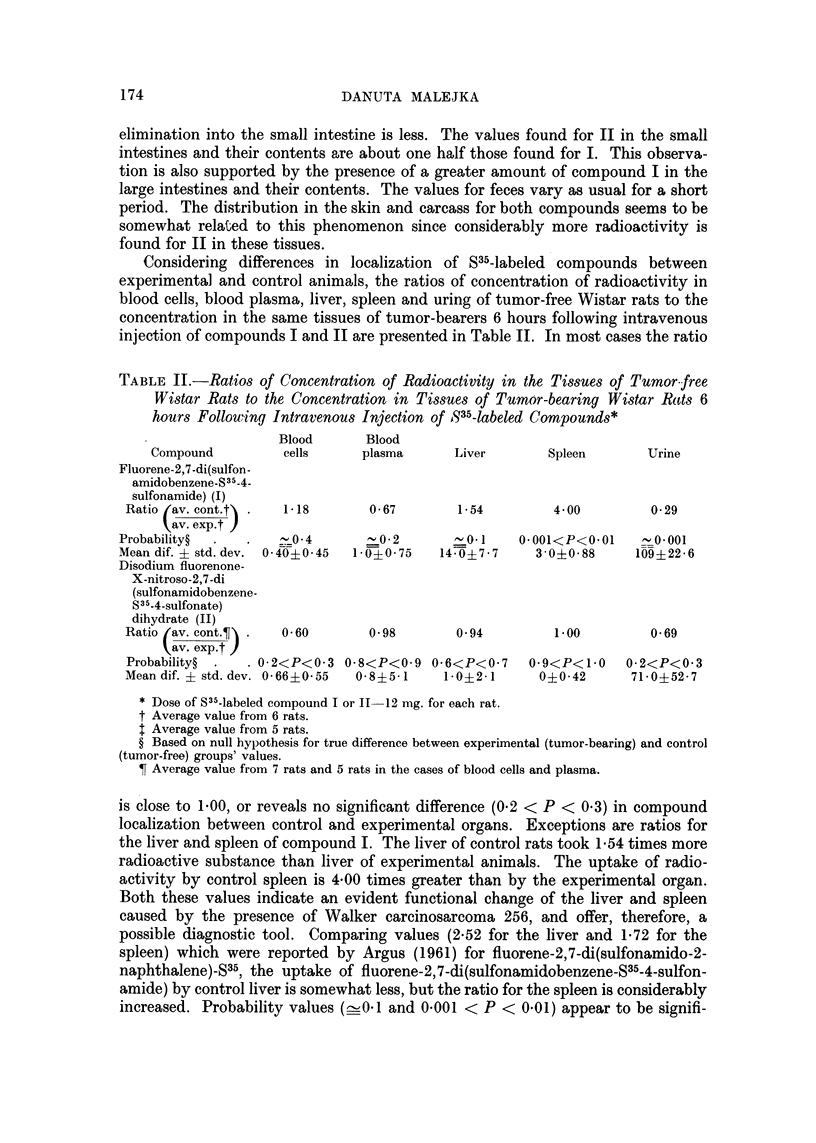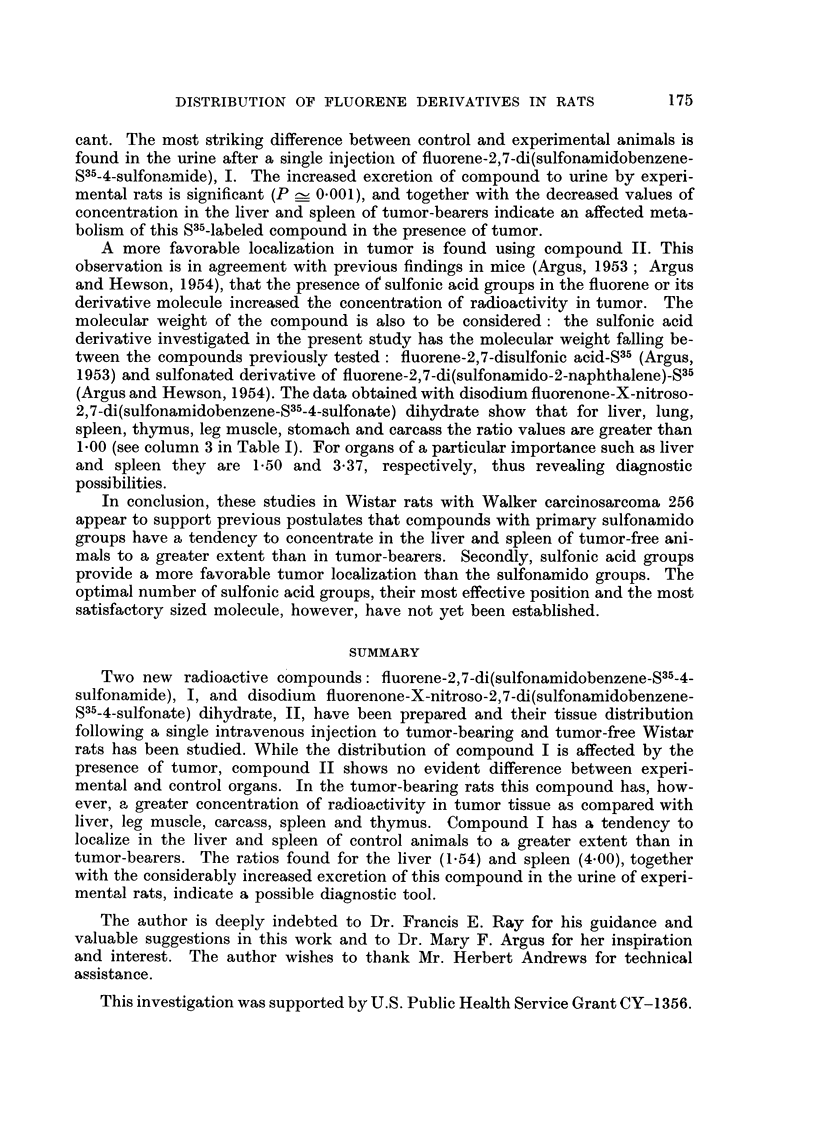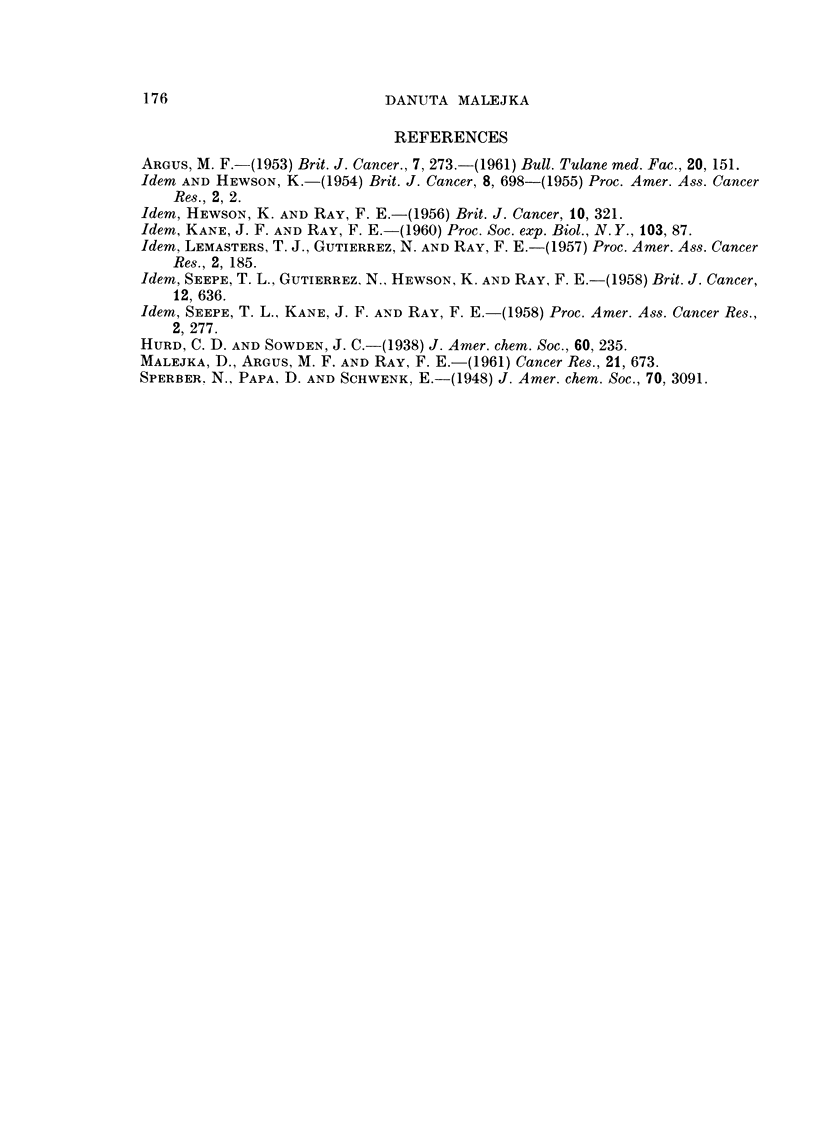# Distribution of Sulfur-35 Labeled Sulfonic Acid Derivatives of Fluorene in Tumour-Bearing and Tumour-Free Rats

**DOI:** 10.1038/bjc.1962.18

**Published:** 1962-03

**Authors:** Danuta Malejka


### DISTRIBUTION OF SULFUR-35 LABELED SULFONIC ACID DERIVATIVES OF FLUORENE IN TUMOR-BEARING AND TUMOR-FREE RATS

DANUTA MALEJKA*

*From the Cancer Research Laboratory, Department of Pharmaceutical Chemistry, University of Florida, Gainesville, Florida.*

Received for publication December 29, 1961

THE use of a radioactive compound as an aid to the diagnosis of an internal cancer would be highly desirable. Previous studies carried out in this laboratory have shown that with certain $S^{35}$-labeled disulfonamido derivatives of fluorene and biphenyl there is an evident difference in the distribution of these compounds between tumor-bearing and tumor-free animal organisms. The most promising results have been obtained with disodium fluorene-2,7-disulfonate-$S^{35}$ (Argus, 1953) and sulfonated derivative of fluorene-2,7-di(sulfonamido-2-naphthalene)-$S^{35}$ (Argus and Hewson, 1954, 1955). Both compounds have been tested in tumor-bearing and tumor-free mice and at each time period studied (2, 8 and 32 hours) the ratios of localization in tumor tissue compared to the liver, spleen and also kidneys, blood and muscle were greatly increased.

On the other hand, an interesting observation has been made with fluorene-2, 7-di(sulfonamido-2-naphthalene)-$S^{35}$ (Argus, Hewson and Ray, 1956) and biphenyl-4,4′-di(sulfonamido-benzene-$S^{35}$-4-sulfonamide) (Argus, Seepe, Gutierrez, Hewson and Ray, 1958), which have been localized in the liver and spleen of tumor-free mice to a greater extent than in tumor-bearers.

Fluorene-2,7-di(sulfonamido-2-naphthalene)-$S^{35}$ showed the same behavior in hamsters with and without transplanted sarcoma (Argus, Lemasters, Gutierrez and Ray, 1957). Furthermore, when the tumors were removed the concentration of radioactivity in the liver and spleen returned to the level found in tumor-free animals. The same perception has been reported for male Wistar rats with Walker carcinosarcoma 256 (Argus, Seepe, Kane and Ray, 1958 ; Argus, 1961).

To determine if, in the presence of tumor, the decreased uptake of fluorene-2, 7-di(sulfonamido-2-naphthalene)-$S^{35}$ by the liver and spleen is a general stress phenomenon, the distribution of this compound has been examined in rats and mice of both sexes after total whole body irradiation of 150 r (Argus, Kane and Ray, 1960) and after cortisone treatment (Malejka, Argus and Ray, 1961). The results of these experiments showed that the presence of tumor in animals is responsible for the differential uptake of disulfonamide by the liver and spleen.

In the present study two new substances were prepared and examined in tumor-bearing and tumor-free Wistar rats in an attempt to find a favorable liver-spleen or tumor localization. These were fluorene-2,7-di(sulfonamidobenzene-$S^{35}$-

* Post-Doctoral Fellow. Permanent address : Department of Pharmaceutical Chemistry, Medical Academy, ul. Grunwaldzka 6, Poznan, Poland.

4-sulfonamide), I, and a compound, the analysis of which corresponds to disodium fluorenone-X-nitroso-2,7-di(sulfonamidobenzene-$S^{35}$-4-sulfonate) dihydrate, II.

I

II

* Radioactive $S^{35}$.

The chemical structures of both compounds are closely related to the group of high molecular weight disulfonamides which were reported on previously. Compound I is an analog of a biphenyl derivative which was tested in mice (Argus, Seepe, Gutierrez, Hewson and Ray, 1958), and compound II was obtained from I by reaction with nitrous anhydride, since this method was reported to be satisfactory in the case of carboxy amides extremely resistant to hydrolysis (Hurd and Sowden, 1938 ; Sperber, Papa and Schwenk, 1948). However, in the described case this resulted, in addition to removal of the amido groups, in a compound the analysis of which corresponds to disodium fluorenone-X-nitroso-2,7-di(sulfonamidobenzene-$S^{35}$-4-sulfonate) dihydrate, indicating supplementary introduction of a nitroso group and oxidation in the 9 position. While this was a more profound change than we had intended, it, nevertheless, seemed of interest to compare the behavior of this less usual derivative with those previously studied.

### MATERIALS AND METHODS

*Fluorene-2,7-disulfonyl chloride-$S^{35}$*.—The procedure described previously (Argus and Hewson, 1954) was employed, but 50 millicuries of sulfur-35 were used with the same quantities of other reactants. The yield of crude material was 25 g. (71 per cent of theory). Recrystallization from toluene gave 6 g. of product (17 per cent of theory), melting at 220–221° ; specific activity was not determined on this intermediate.

*Fluorene-2,7-di(sulfonamidobenzene-$S^{35}$-4-sulfonamide), I*.—Sulfanilamide, 14·2 g. (0·082 moles) was dissolved in 120 ml. methyl ethyl ketone at 55–60°. The solution was stirred and maintained at 60° throughout the addition (15 minutes) of fluorene-2,7-disulfonyl chloride-$S^{35}$, 6 g. (0·016 moles), after which the temperature of the water bath was raised to and maintained at 95° for 7 hours. The white, thick reaction mixture was allowed to cool to room temperature, the precipitate was collected, washed free of sulfanilamide hydrochloride with hot distilled water, and dried over phosphorus pentoxide. The yield of crude material was 10 g. (95·41 per cent of theory), melting point 280–282°. Recrystallization from dioxane and then 95 per cent ethanol gave 6 g. (57·25 per cent of theory) of white product melting at 290–292° and having a specific activity of 7100 disintegrations per second per mg. (0·19 μc per mg.).

*Analysis*.—Calculated for $C_{25}H_{22}N_4S_4O_8$ : C, 47·32 ; H, 3·49 ; N, 8·82 ; S, 20·21.

Found : C, 47·55 ; H, 3·48 ; N, 8·92 ; S, 20·39.

*Disodium fluorenone-X-nitroso-2,7-di(sulfonamidobenzene-$S^{35}$-4-sulfonate) dihydrate, II*.—Fluorene-2,7-di(sulfonamido-benzene-$S^{35}$-4-sulfonamide), 4·5 g. (0·007 moles), was suspended in 35 ml. glacial acetic acid. The suspension was treated at 5° with nitrous anhydride (generated by the action of arsenic trioxide, 30 g. (0·15 moles) and concentrated nitric acid 29 g. (0·45 moles)) until the material dissolved (about 30 minutes). A lively evolution of nitrogen occurred almost immediately after introduction of the nitrous anhydride. The reaction mixture was left for an hour in ice, and then at room temperature under a well ventilated hood for at least 4 hours to remove the excess of gas from the solution. To the light yellow acid solution (filtered if not quite clear), a saturated solution of sodium acetate was added slowly with stirring to bring the pH to about 9. Thus, the yellow sodium salt of sulfonic acid was precipitated and collected. The yield was 1·5 g. (30·61 per cent of theory) with decomposition point about 300°. The double extraction of impurities with absolute ethanol gave 1 g. (20·41 per cent of theory) of insoluble residue melting at 283° and having a specific activity of 5300 disintegrations per second per mg. (0·14 μc per mg.). It retained two molecules of water very tenaciously.

*Analysis*.—Calculated for $C_{25}H_{19}N_3S_4O_{14}Na_2$: C, 39·53; H, 2·52; N, 5·53; S, 16·88; Na, 6·05.

Found : C, 39·15 ; H, 2·62 ; N, 5·39 ; S, 15·96 ; Na, 5·82.

The sodium salt shaken with Amberlite IR–120 gave the free sulfonic acid derivative as a glass, which decomposed at 225°. It was not further examined, but was converted into the mono-*p*-toluidine salt, which melted at 235°. This retained one molecule of water.

*Analysis*.—Calculated for $C_{32}H_{28}N_4S_4O$ : C, 47·75 ; H, 3·51 ; N, 6·96 ; S, 15·94.

Found : C, 47·91 ; H, 3·99 ; N, 7·30 ; S, 15·25.

*Animal experiments*.—A total of 24 male Wistar rats, weanlings, was used ; 11 were employed for the subcutaneous transplantation of Walker carcinosarcoma 256, now in its 115th generation transplant in this laboratory ; 13 tumor-free rats served as control groups. When the tumors were 10–12 days old, and averaged 12 g. in weight, the radioactive compounds were administered by tail vein injections. Each rat was given 1·2 ml. 0·05 N sodium hydroxide containing 12 mg. fluorene-2,7-di(sulfonamidobenzene-$S^{35}$-4-sulfonamide), I, or disodium fluorenone-X-nitroso-2, 7-di(sulfonamidobenzene-$S^{35}$-4-sulfonate) dihydrate, II, and then placed in an individual metabolism cage and killed 6 hours following administration of the compounds. Concentration and per cent recovery of radioactive material in tissues and excreta of rats were determined by methods previously described (Argus, Kane and Ray, 1960). Radioactivity measurements were made in the gas-flow GM counter of the low background automatic sample changer, Model C115, with decade scaler Model 181A and printer Model C111B, from the Nuclear Chicago Corporation. The efficiency of the instrument was 25·8 per cent.

### RESULTS AND DISCUSSION

The complete tissue distribution of fluorene-2,7-di(sulfonamidobenzene-$S^{35}$-4-sulfonamide), I, and disodium fluorenone-X-nitroso-2,7-di(sulfonamidobenzene-$S^{35}$-4-sulfonate) dihydrate, II, between tumor-bearing and tumor-free Wistar rats, was compared. Since previous experiments have shown a 6 hours time interval to be relatively effective for the distribution of disulfonamido derivatives of fluorene (Argus, Kane and Ray, 1960 ; Malejka, Argus and Ray, 1961), the present investigation was conducted under the same condition. The details of these studies for compounds I and II are shown in Table I. Both concentrations, expressed as μg.

TABLE I.—*Distribution of Radioactivity in Tumor-bearing and Tumor-free Wistar Rats 6 Hours following Intravenous Injection of Fluorene-2,7-di(Sulfonamido-benzene-$S^{35}$-4-sulfonamide), I, and Disodium Fluorenone-X-Nitroso-2,7-di(sulfonamidobenzene-$S^{35}$-4-sulfonate) Dihydrate, II**

| Tissue | Tumor-bearing rats | | | | Tumor-free rats | | | |
|---|---|---|---|---|---|---|---|---|
| | Compound I† | | Compound II‡ | | Compound I‡ | | Compound II§ | |
| | μg./g. | Per cent | μg./g. | Per cent | μg./g. | Per cent | μg./g. | Per cent |
| Blood cells¶ | 2 | 0·08 | 1 | 0·06 | <3† | 0·08 | <1† | 0·02 |
| Blood plasma¶ | 3 | 0·10 | 40 | 1·58 | 2 | 0·06 | 39† | 1·17 |
| Liver | 26 | 1·27 | 18 | 1·09 | 40 | 2·00 | 17 | 0·73 |
| Lung | 5 | 0·03 | 26 | 0·16 | 9 | 0·05 | 24 | 0·12 |
| Spleen | 1 | <0·01 | 8 | 0·08 | 4 | 0·01 | 8 | 0·03 |
| Kidney | 15 | 0·12 | 45 | 0·46 | 16 | 0·15 | 41 | 0·37 |
| Thymus | 3 | <0·01 | 5 | 0·01 | 2 | <0·01 | 9 | 0·02 |
| Skin | 24 | 4·84 | 51 | 10·95 | 26 | 5·13 | 53 | 8·47 |
| Leg muscle | 2 | 0·02 | 9 | 0·07 | 2 | 0·02 | 13 | 0·10 |
| Stomach + contents | 21 | 0·32 | 20 | 0·26 | 10 | 0·12 | 40 | 0·45 |
| Small intestine + contents | 503 | 24·90 | 272 | 13·64 | 572 | 21·87 | 247 | 11·99 |
| Large intestine + contents | 1412 | 67·08 | 970 | 53·93 | 1277 | 68·30 | 1078 | 55·19 |
| Carcass | 4 | 1·75 | 9 | 4·21 | 3 | 1·72 | 13 | 5·57 |
| Urine¶ | 153 | 1·27 | 229 | 1·95 | 44 | 0·37 | 158 | 1·33 |
| Feces | 407 | 0·30 | 53† | 0·06 | 9 | 0·03 | 252‡ | 1·52 |
| Tumor | 15‖ | 0·92 | 27 | 3·02 | — | — | — | — |
| Total | | 103·02 | | 91·53 | | 99·92 | | 87·08 |

* Dose of $S^{35}$-labeled compound I or II—12 mg. for each rat.
† Average value from 5 rats.
‡ Average value from 6 rats.
§ Average value from 7 rats.
‖ Average value from 4 rats.
¶ Concentration in μg. compound/ml. ; others expressed in μg. compound/g. tissue.

of compound per g. tissue or ml. blood or urine, and percentages of administered dose recovered are given.

Differences between the distribution of the two substances are evident. While the values for blood cells and blood plasma for fluorene-2,7-di(sulfonamidobenzene-$S^{35}$-4-sulfonamide) are relatively small and nearly the same, there is a great difference between these values for the second compound, the concentration in plasma being 40 times greater than in red blood cells. This is a key to the rate of distribution of these compounds in the animal body. Compound II is absorbed from the blood at a slower rate than I. The filtration of compound II through the liver is slower than I and is not changed by the presence of tumor. Consequently, elimination into the small intestine is less. The values found for II in the small intestines and their contents are about one half those found for I. This observation is also supported by the presence of a greater amount of compound I in the large intestines and their contents. The values for feces vary as usual for a short period. The distribution in the skin and carcass for both compounds seems to be somewhat related to this phenomenon since considerably more radioactivity is found for II in these tissues.

Considering differences in localization of $S^{35}$-labeled compounds between experimental and control animals, the ratios of concentration of radioactivity in blood cells, blood plasma, liver, spleen and uring of tumor-free Wistar rats to the concentration in the same tissues of tumor-bearers 6 hours following intravenous injection of compounds I and II are presented in Table II. In most cases the ratio

TABLE II.—*Ratios of Concentration of Radioactivity in the Tissues of Tumor-free Wistar Rats to the Concentration in Tissues of Tumor-bearing Wistar Rats 6 hours Following Intravenous Injection of $S^{35}$-labeled Compounds**

| Compound | Blood cells | Blood plasma | Liver | Spleen | Urine |
|---|---|---|---|---|---|
| Fluorene-2,7-di(sulfonamidobenzene-$S^{35}$-4-sulfonamide) (I) | | | | | |
| Ratio $\left(\frac{\text{av. cont.†}}{\text{av. exp.†}}\right)$ . | 1·18 | 0·67 | 1·54 | 4·00 | 0·29 |
| Probability§ . . | ≃0·4 | ≃0·2 | ≃0·1 | $0·001<P<0·01$ | ≃0·001 |
| Mean dif. ± std. dev. | 0·40±0·45 | 1·0±0·75 | 14·0±7·7 | 3·0±0·88 | 109±22·6 |
| Disodium fluorenone-X-nitroso-2,7-di (sulfonamidobenzene-$S^{35}$-4-sulfonate) dihydrate (II) | | | | | |
| Ratio $\left(\frac{\text{av. cont.¶}}{\text{av. exp.†}}\right)$ . | 0·60 | 0·98 | 0·94 | 1·00 | 0·69 |
| Probability§ . . | $0·2<P<0·3$ | $0·8<P<0·9$ | $0·6<P<0·7$ | $0·9<P<1·0$ | $0·2<P<0·3$ |
| Mean dif. ± std. dev. | 0·66±0·55 | 0·8±5·1 | 1·0±2·1 | 0±0·42 | 71·0±52·7 |

* Dose of $S^{35}$-labeled compound I or II—12 mg. for each rat.
† Average value from 6 rats.
‡ Average value from 5 rats.
§ Based on null hypothesis for true difference between experimental (tumor-bearing) and control (tumor-free) groups' values.
¶ Average value from 7 rats and 5 rats in the cases of blood cells and plasma.

is close to 1·00, or reveals no significant difference ($0·2 < P < 0·3$) in compound localization between control and experimental organs. Exceptions are ratios for the liver and spleen of compound I. The liver of control rats took 1·54 times more radioactive substance than liver of experimental animals. The uptake of radioactivity by control spleen is 4·00 times greater than by the experimental organ. Both these values indicate an evident functional change of the liver and spleen caused by the presence of Walker carcinosarcoma 256, and offer, therefore, a possible diagnostic tool. Comparing values (2·52 for the liver and 1·72 for the spleen) which were reported by Argus (1961) for fluorene-2,7-di(sulfonamido-2-naphthalene)-$S^{35}$, the uptake of fluorene-2,7-di(sulfonamidobenzene-$S^{35}$-4-sulfonamide) by control liver is somewhat less, but the ratio for the spleen is considerably increased. Probability values (≃0·1 and $0·001 < P < 0·01$) appear to be significant. The most striking difference between control and experimental animals is found in the urine after a single injection of fluorene-2,7-di(sulfonamidobenzene-$S^{35}$-4-sulfonamide), I. The increased excretion of compound to urine by experimental rats is significant ($P \cong 0{\cdot}001$), and together with the decreased values of concentration in the liver and spleen of tumor-bearers indicate an affected metabolism of this $S^{35}$-labeled compound in the presence of tumor.

A more favorable localization in tumor is found using compound II. This observation is in agreement with previous findings in mice (Argus, 1953 ; Argus and Hewson, 1954), that the presence of sulfonic acid groups in the fluorene or its derivative molecule increased the concentration of radioactivity in tumor. The molecular weight of the compound is also to be considered : the sulfonic acid derivative investigated in the present study has the molecular weight falling between the compounds previously tested : fluorene-2,7-disulfonic acid-$S^{35}$ (Argus, 1953) and sulfonated derivative of fluorene-2,7-di(sulfonamido-2-naphthalene)-$S^{35}$ (Argus and Hewson, 1954). The data obtained with disodium fluorenone-X-nitroso-2,7-di(sulfonamidobenzene-$S^{35}$-4-sulfonate) dihydrate show that for liver, lung, spleen, thymus, leg muscle, stomach and carcass the ratio values are greater than 1·00 (see column 3 in Table I). For organs of a particular importance such as liver and spleen they are 1·50 and 3·37, respectively, thus revealing diagnostic possibilities.

In conclusion, these studies in Wistar rats with Walker carcinosarcoma 256 appear to support previous postulates that compounds with primary sulfonamido groups have a tendency to concentrate in the liver and spleen of tumor-free animals to a greater extent than in tumor-bearers. Secondly, sulfonic acid groups provide a more favorable tumor localization than the sulfonamido groups. The optimal number of sulfonic acid groups, their most effective position and the most satisfactory sized molecule, however, have not yet been established.

### SUMMARY

Two new radioactive compounds: fluorene-2,7-di(sulfonamidobenzene-$S^{35}$-4-sulfonamide), I, and disodium fluorenone-X-nitroso-2,7-di(sulfonamidobenzene-$S^{35}$-4-sulfonate) dihydrate, II, have been prepared and their tissue distribution following a single intravenous injection to tumor-bearing and tumor-free Wistar rats has been studied. While the distribution of compound I is affected by the presence of tumor, compound II shows no evident difference between experimental and control organs. In the tumor-bearing rats this compound has, however, a greater concentration of radioactivity in tumor tissue as compared with liver, leg muscle, carcass, spleen and thymus. Compound I has a tendency to localize in the liver and spleen of control animals to a greater extent than in tumor-bearers. The ratios found for the liver (1·54) and spleen (4·00), together with the considerably increased excretion of this compound in the urine of experimental rats, indicate a possible diagnostic tool.

The author is deeply indebted to Dr. Francis E. Ray for his guidance and valuable suggestions in this work and to Dr. Mary F. Argus for her inspiration and interest. The author wishes to thank Mr. Herbert Andrews for technical assistance.

This investigation was supported by U.S. Public Health Service Grant CY–1356.

### REFERENCES

ARGUS, M. F.—(1953) *Brit. J. Cancer.*, **7**, 273.—(1961) *Bull. Tulane med. Fac.*, **20**, 151.
*Idem* AND HEWSON, K.—(1954) *Brit. J. Cancer*, **8**, 698—(1955) *Proc. Amer. Ass. Cancer Res.*, **2**, 2.
*Idem*, HEWSON, K. AND RAY, F. E.—(1956) *Brit. J. Cancer*, **10**, 321.
*Idem*, KANE, J. F. AND RAY, F. E.—(1960) *Proc. Soc. exp. Biol., N.Y.*, **103**, 87.
*Idem*, LEMASTERS, T. J., GUTIERREZ, N. AND RAY, F. E.—(1957) *Proc. Amer. Ass. Cancer Res.*, **2**, 185.
*Idem*, SEEPE, T. L., GUTIERREZ, N., HEWSON, K. AND RAY, F. E.—(1958) *Brit. J. Cancer*, **12**, 636.
*Idem*, SEEPE, T. L., KANE, J. F. AND RAY, F. E.—(1958) *Proc. Amer. Ass. Cancer Res.*, **2**, 277.
HURD, C. D. AND SOWDEN, J. C.—(1938) *J. Amer. chem. Soc.*, **60**, 235.
MALEJKA, D., ARGUS, M. F. AND RAY, F. E.—(1961) *Cancer Res.*, **21**, 673.
SPERBER, N., PAPA, D. AND SCHWENK, E.—(1948) *J. Amer. chem. Soc.*, **70**, 3091.